# A Subband-Specific Deconvolution Model for MTF Improvement in CT

**DOI:** 10.1155/2017/2193635

**Published:** 2017-10-25

**Authors:** Seokmin Han, Kihwan Choi, Sang Wook Yoo

**Affiliations:** ^1^Korea National University of Transportation, Chungju, Republic of Korea; ^2^Samsung Electronics, Suwon-si, Kyunggi-do 443-742, Republic of Korea

## Abstract

The purpose of this research is to achieve uniform spatial resolution in CT (computed tomography) images without hardware modification. The main idea of this study is to consider geometry optics model, which can provide the approximate blurring PSF (point spread function) kernel, which varies according to the distance from X-ray tube to each pixel. The FOV (field of view) was divided into several band regions based on the distance from X-ray source, and each region was deconvolved with different deconvolution kernels. Though more precise calculation for the PSF for deconvolution is possible as the number of subbands increases, we set the number of subbands to 11. 11 subband settings seem to be a balancing point to reduce noise boost, while MTF (modulation transfer function) increase still remains. As the results show, subband-wise deconvolution makes image resolution (in terms of MTF) relatively uniform across the FOV. The results show that spatial resolution in CT images can be uniform across the FOV without using additional equipment. The beauty of this method is that it can be applied to any CT system as long as we know the specific system parameters and determine the appropriate PSF for deconvolution maps of the system. The proposed algorithm shows promising result in improving spatial resolution uniformity while avoiding the excessive noise boost.

## 1. Introduction

Image quality is of primary concern in diagnostic and screening imaging. Among many elements that affect the image quality, resolution is one of the most concerned. For the early detection of diseases, high spatial resolution imaging is very important. The goal of high spatial resolution is to look into small structures such as airways, arteries, and lesions as well as to detect small changes, so that prevention and intervention can be made earlier. The spatial resolution of a CT image is affected by a number of factors including the focal spot size of X-ray tube, detector size, scattering, magnification, the number of projections per rotation, and the reconstruction process [1]. The contribution of each factor is hard to determine separately; however, they can be lumped into PSF which determines the final measured resolution in the images. It is also known that the resolution in the FOV decreases as the distance from isocenter increases, which results in spatial variation of resolution. The spatial variation of resolution can be also considered as spatial variation of PSF. This variation affects the image quality, which sometimes can cause mistakes in inspection, such as overlooking a calcification at the periphery region. Because of the spatial variation of resolution, the patient table is steered so that the heart of the imaged patient should be located at the isocenter. If the problem of the spatial variation of resolution is alleviated, it can be beneficial for improving the imaging workflow.

Different techniques have been used to overcome this problem. They can be divided into two categories. One category is to employ specially designed hardware such as high-resolution detector [2], focal-spot wobbling on the X-ray tube [3, 4], or aperture collimator to refine the focus of X-ray source [5]. The other category is to apply model-based computation such as optics model [6–10] or system model [11–15]. Each method has its own advantage. However, the idea of recovering the inherent spatial resolution of CT images using the knowledge of the system blurring PSF and its deconvolution can be practical in that it does not require any additional hardware modification. The main idea of this study is to consider geometrical optics model, which can provide the approximation of the blurring PSF kernel dependent on the distance from X-ray tube to each pixel. Based on the approximation, we can deconvolve each pixel to recover the inherent spatial resolution of CT images.

While previous methods generally employ iterative reconstruction or forward projection to combine PSF with reconstruction, the authors' method is an analytical method like FBP (filtered backprojection). In the authors' previous method [16], we had to increase the number of measurements to precisely estimate the variation of the blurring PSF. In the proposed method, we estimate the variation of the blurring PSF based on the fixed number of measurements. And it should be also noted that the purpose of the previous method was to enhance MTF at the isocenter, not to make MTF uniform across the FOV. The proposed method is an extended method of the previous method [16] in that it does not require PSF measurement in every subband and can be applied to alleviate the problem of spatial variation of reconstructed image resolution in CT.

## 2. Methods and Materials

To improve the resolution of reconstructed CT image, deconvolution procedure was employed. Backprojection is calculating the contribution of each voxel of the imaged structure to the measured data. For the calculation, the images of all views are backprojected and accumulated. The backprojection of each view was separated into several band regions according to the distance from the X-ray source, and each of the band regions was deconvolved with a corresponding deconvolution kernel. Because X-ray source moves along a scanning trajectory, the distance from the source to an arbitrary point in the FOV varies. Therefore, each point in the FOV goes through plural band regions. As a result, each point in the reconstructed image is deconvolved with plural corresponding deconvolution kernels, which can be regarded as a function dependent on the distance from isocenter.

### 2.1. The Concept of Subband Deconvolution of FOV

**Figure 1:**
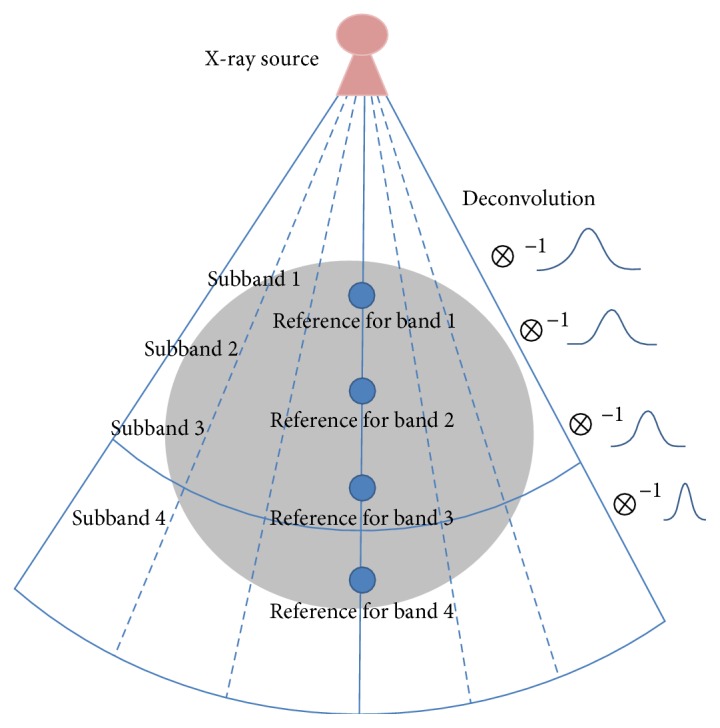
If the distance from X-ray source to ROI is close, the divergence of beam becomes wide.

In fanbeam case, X-ray beam diverges as it propagates. The divergence depends on the distance from X-ray source to the measurement point, which is the ROI (region of interest). If it is far from the source, and close to the detector, the beam divergence becomes relatively narrow. We measured PSF bandwidth at five locations using 1 mm cylindrical rods of aluminium, and the Gaussian fit to each PSF was used as the deconvolution kernel. Thus, the kernel width was experimentally determined to approximate the PSF of the center of each subband. The precise measurement procedure is explained in the next section. For deconvolution, MATLAB built-in *deconvreg* function was applied. This concept is shown in Figure 1.

In Figure 1, we separated the FOV into three subbands. Each kernel corresponding to each subband was used to deconvolve the backprojection for each view. As illustrated in Figure 1, relatively wide kernel width corresponds to the subband close to the X-ray source, while relatively narrow kernel width corresponds to the subband far from the X-ray source.

### 2.2. The Calculation of Deconvolution Kernel Width

**Figure 2:**
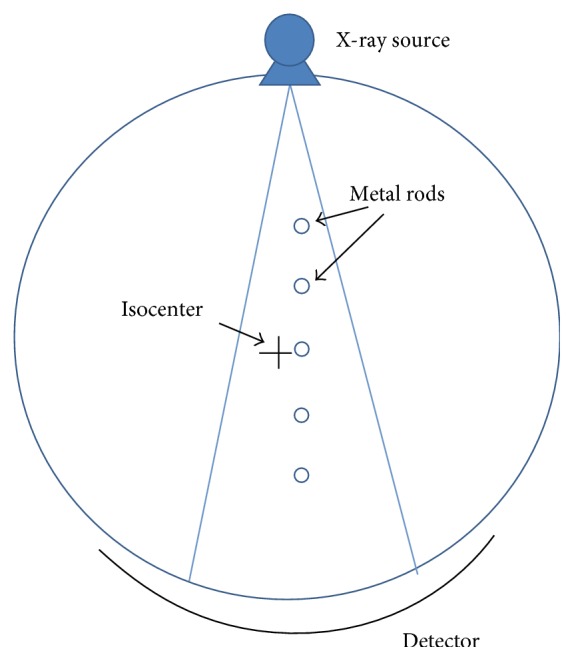
Locations to measure the kernel width in FOV.

We deconvolved each subband in backprojected image for each view. To determine the kernel width values for each subband, we put cylindrical rods of aluminium in the FOV to measure the amount of blur, as can be seen in Figure 2. We measured the blur at five locations (0 cm, 7 cm, 14 cm, −7 cm, and −14 cm from isocenter). The rods were scanned at 120 kV, 100 mAs, and 60 rpm (rotations per minute), which provides 1440 views per rotation. The detector cell size was 1.09 mm, and each detector row has 912 cells. Ram-Lak filter was used for filtering the acquired sinogram, and slice thickness was 6 mm. Each location of the rods was on the line which connects the X-ray source and isocenter. The metal rod was scanned one by one at each location and produced five sinograms. Because we have five sinograms, we could have five acquisitions of data at each view. With the five acquisitions of data, we could estimate the amount of blur and the kernel width according to the distance from X-ray tube to each rod, based on the knowledge about the shape of the aluminium rod. From the estimated blur and kernel width, we obtained PSF. The PSFs were fitted to Gaussian functions, and we estimated the kernel width of the Gaussian functions. The estimated kernel width values were 0.1, 0.4, 0.95, 1.0, and 1.1. After the estimation of the kernel width at each location, we calculate the rational function expressed in (1) to approximate the relation between the kernel width and the distance from the X-ray source to the rod.

(1) $$\begin{array}{c} \sigma =\frac{ax^{2}+bx+c}{dx+e}, \end{array}$$

where *x* is the distance from the X-ray source, *σ* is the kernel width, and *a*, *b*, *c*, *d*, and *e* are coefficients. To solve (1), at least five pairs of kernel width and distance from X-ray source are required. From (1), we could estimate deconvolution kernel width at any location in FOV. Different from the previous research of the authors [16], we applied model fit to the measurement in this research for comfortability in experiments and practical use, rather than measuring the blur at many locations across the FOV.

### 2.3. Subband Deconvolution-Based Backprojection

**Figure 3:**
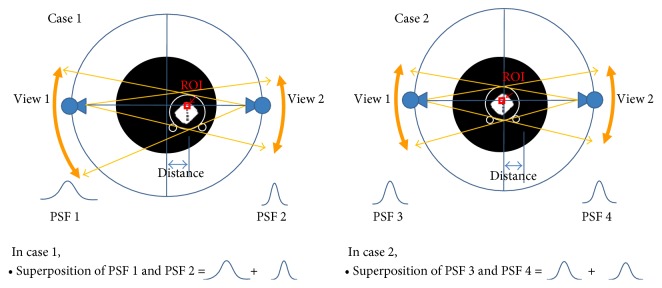
The closer is the distance from the X-ray source to ROI, the wider is the divergence of beam.

The subband that corresponds to a specific ROI varies, because X-ray source rotates along a scanning trajectory.  Figure 3 shows the concept of the distance-divergence relation as the X-ray source rotates.

In case 1 of Figure 3, ROI is apart from isocenter, which makes the corresponding PSF varies in width as the distance from X-ray source varies. In case 2, ROI is at the isocenter, and the corresponding PSF does not vary. If we divide the FOV into several subbands, a specific ROI moves through plural subbands as X-ray source rotates along a scanning trajectory. This process is repeated until all views are backprojected and accumulated. In this way, each point in the FOV is assigned with a corresponding superposed kernel. In this research, we set the number of subbands to 11, which was found experimentally to give the balance between the overshoot and MTF improvement.

This process can be summarized by the following:

(2) $$\begin{array}{c} f\left(x,y\right)=\int_{0}^{2\pi }\left[\overset{n}{\underset{i=1}{\sum }}d_{i}\left[L^{-2}\int_{-\gamma_{m}}^{\gamma_{m}}q\left(\gamma ,\beta \right)h\left(\gamma^{\prime }-\gamma \right)D\cos\left(\gamma \right)d\gamma \right]\right]d\beta , \end{array}$$

where *f*(*x*, *y*) is the image, *n* is the number of subbands (in this research, we set this number to 11), *d*_*i*_ is the deconvolution function for *i*th subband, *L* is the distance from the X-ray source to the point of reconstruction (*x*, *y*), *β* is the projection angle, *γ* is the detector angle, *γ*_*m*_ is the maximum detector angle from the centerline of the detector, *q*(*γ*, *β*) is the projection sample, *γ*′ is the detector angle of the ray that passes through (*x*, *y*), *h*(*γ*′ − *γ*) is the filter, and *D* is the distance between X-ray source and isocenter. Except the deconvolution function *d*_*i*_ and the summation of the deconvolved subbands, the equation equals to the ordinary FBP [17].

## 3. Experiments and Results

We acquired the sinogram data of Helios QA phantom using BodyTom CT scanner (NeuroLogica Inc., Danvers, MA), moving the phantom 6 mm, 47 mm, 79 mm, 127 mm, and 171 mm from the isocenter. The scanning parameters used for the CT scanner were 120 kV and 100 mAs with 1440 views per rotation (acquired over 360°) at 60 rpm (rotation per minute). The detector cell size was 1.09 mm, and each detector row has 912 cells. By default, Ram-Lak filter was used for filtering the acquired sinogram, and slice thickness was 6 mm. In addition, the filtered sinogram was deconvolved with appropriate Gaussian kernels. For the deconvolution, we divided FOV to 11 subbands and applied MATLAB built-in *deconvreg* function to each subband, to which appropriate Gaussian filter was assigned.

**Figure 4:**
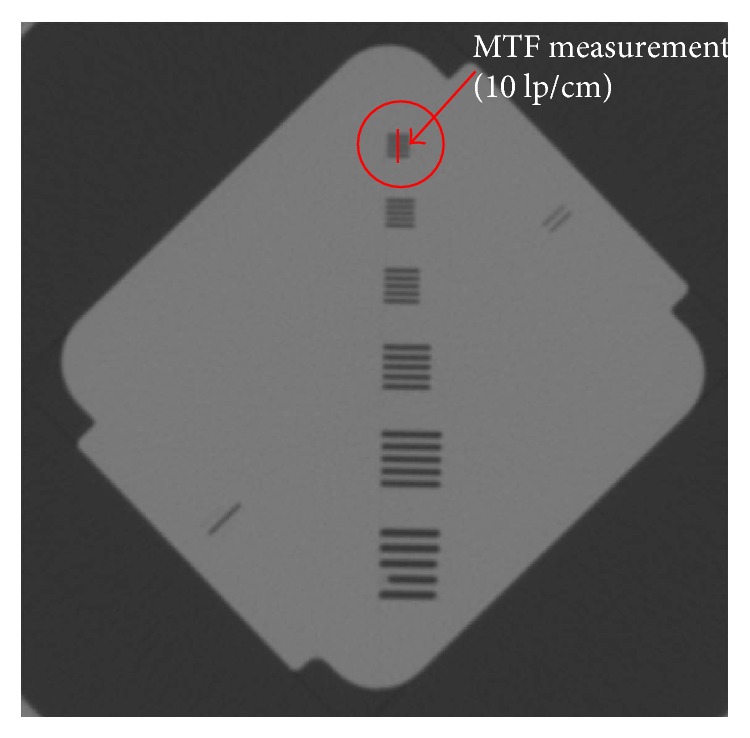
Helios image for describing MTF measurement region.

The subband deconvolution was applied to each Helios QA phantom sinogram. To test the feasibility of the proposed method, about 25 images have been reconstructed. In Figure 4, the region for MTF measurement is described. The region pointed by red arrow corresponds to 10 lp/cm.

**Figure 5:**
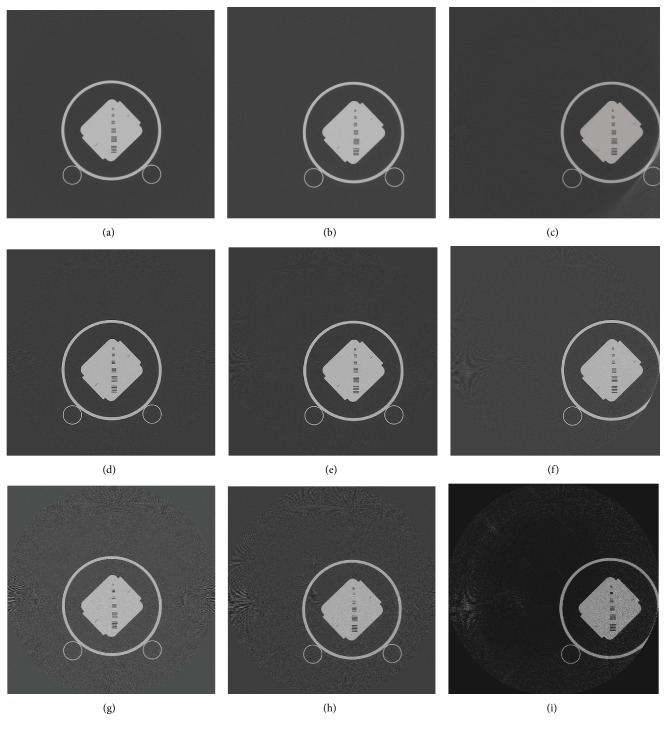
Helios images. (a) 6 mm from isocenter (FBP), (b) 47 mm from isocenter (FBP), (c) 127 mm from isocenter (FBP), (d) 6 mm from isocenter (sharp FBP), (e) 47 mm from isocenter (sharp FBP), (f) 127 mm from isocenter (sharp FBP), (g) 6 mm from isocenter (11 subbands), (h) 47 mm from isocenter (11 subbands), (i) 127 mm from isocenter (11 subbands).

In Figure 5, the reconstructed Helios QA phantom images at 6 mm, 47 mm, and 127 mm from the isocenter are shown. For comparison, normal FBP images, sharpened FBP images, and 11 subband-reconstructed images are presented.

**Figure 6:**
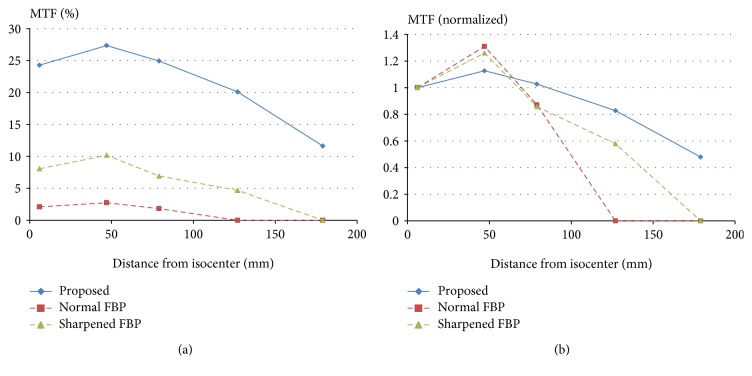
(a) MTF curves at 10 lp/cm as the phantom moves from isocenter. (b) MTF curves normalized by each of the MTF values at 6 mm from isocenter.

The performance was measured in terms of MTF of the reconstructed image. In Figure 6, the MTF values measured at 10 lp/cm are shown as MTF curves. In Figure 6(a), the MTF values of each method are drawn for comparison. Absolute values of them have different ranges. Absolute value of the proposed method has a range of almost 20, which is much higher than the other methods. Thus, we also present the MTF values normalized by each of the MTF values at 6 mm from isocenter in Figure 6(b). Seeing the result in Figure 6, it seems that MTF of the proposed method remains relatively stable at each position.

**Figure 7:**
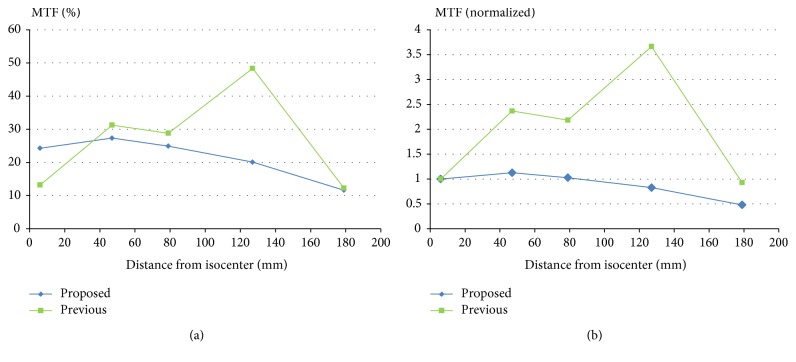
The previous method of the author [16] is compared with the proposed method. (a) MTF curves at 10 lp/cm as the phantom moves from isocenter. (b) MTF curves normalized by each of the MTF values at 6 mm from isocenter.

In Figure 7, the proposed method is compared with the previous method of the author [16]. In the previous method, the subband-specific kernel width is not estimated using a model, but measured at each subband. And it should be also noted that the purpose of the previous method was to enhance MTF at isocenter, not to make MTF uniform across the FOV. As can be seen in Figure 7, the proposed method shows much more uniform and stable MTF.

**Figure 8:**
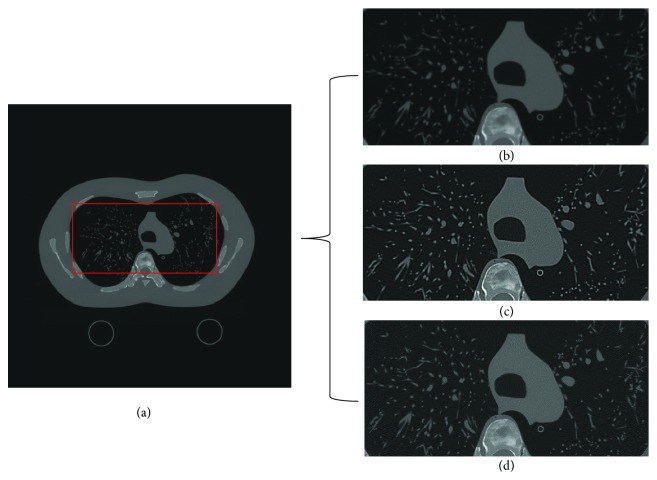
Lung phantom images. (a) The region to be compared. (b) FBP, (c) sharp FBP, and (d) 11 subband-reconstructed images.

In Figure 8, the reconstructed lung phantom images are shown for a simulation of clinical image. For comparison, normal FBP image, sharp reconstructed image, and 11 subband-reconstructed images are shown. As can be seen in Figure 8, the proposed method does not show much overshoot while maintaining relatively sharp edge.

**Figure 9:**
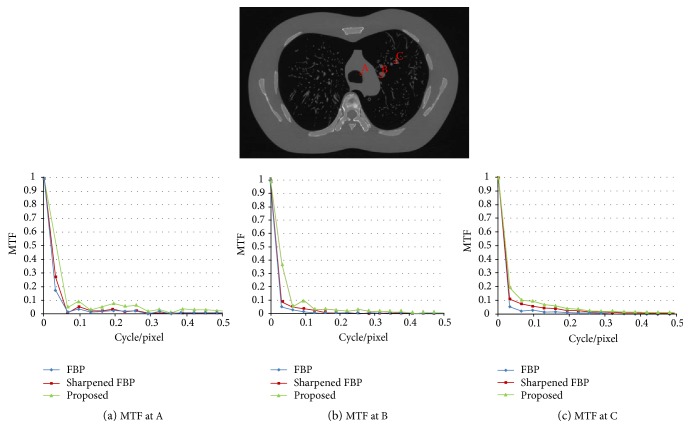
(a) MTF curve at A, (b) MTF curve at B, and (c) MTF curve at C.

Though the material of lung phantom is close to soft tissue in human body and it is not used for MTF measurement, we tried to measure MTF values at several positions of the lung phantom image to show the effect of the proposed method. MTF curves measured at several points are presented in Figure 9. As can be seen in Figure 9, MTF curves of the proposed method show higher MTF values than normal MTF or sharpened MTF at relatively high-frequency region. Particularly, the location of the intersect between MTF curves of the proposed method and 0.1 MTF seems to remain relatively stable, near 0.05 cycles/pixel. This shows that the proposed method seems to work even in soft tissue region.

## 4. Discussion

The proposed method shows a promising result in that the reconstructed image can have relatively uniform resolution across the FOV, in terms of MTF. Another advantage of the proposed method is that it is an analytical method, without thresholding or regularization. The proposed method implicitly assigns appropriate kernels to each pixel. Because we know that X-ray beam diverges as it propagates, we apply wide width PSF to the subband region close to X-ray tube for deconvolution and narrow width PSF to the far subband region. This process is done view by view, and the resultant effect of deconvolution kernel width for each pixel is the summation of the deconvolution PSF kernels for that pixel. Thus, we can provide different deconvolution PSF with different location in the FOV. The resultant image can be considered as deconvolved by location-dependent filters. Therefore, we can guess that it may be better to have many subbands to restore the signal. Though more precise calculation for the PSF for deconvolution is possible as the number of subbands increases, we set the number of subbands to 11 experimentally. 11 subband settings seem to be the balancing point to reduce noise boost, while MTF increase still remains.

As the results show, subband-wise deconvolution makes image resolution (in terms of MTF) relatively uniform across the FOV. MTF values were kept around 20% from 6 mm to 127 mm apart from the isocenter. This can be very beneficial in clinical situation, because relatively uniform resolution alleviates the necessity to move the patient table so that the imaging region should be near the isocenter. If the location-dependent filter completely restores the signal, MTF values may be equal across the FOV. For complete restoration of the signal, denser PSF measurements across the FOV may be requisite. Currently, we assumed that the same PSF width can be applied in a subband region. However, the subband can be also divided in angular direction to make the location-dependent filter more accurate.

## 5. Conclusion

The results show that spatial resolution in CT images can be uniform across the FOV without using additional equipment. The beauty of this method is that it can be applied to any CT system as long as we know the specific system parameters and determine the appropriate deconvolution PSF maps of the system. The proposed algorithm shows promising result in improving spatial resolution uniformity while avoiding the excessive noise boost. This technique can possibly improve the detection and quantification of small structures in the heart, lung, and brain images and improve workflow efficiency by alleviating the necessity to move the patient table so that the imaging region should be near the isocenter.
